# Supplementary material for: Multiple-testing corrections in case-control studies using identity-by-descent segments
Source: bioRxiv. 2025 Jul 7:2025.07.03.663057. Preprint. [Version 1] doi: 10.1101/2025.07.03.663057 (PMC12265659; doi:10.1101/2025.07.03.663057)
Supplement: 1 [file NIHPP2025.07.03.663057V1-supplement-1.pdf]

bioRxiv preprint doi: https://doi.org/10.1101/2025.07.03.663057; this version posted July 7, 2025. The copyright holder for this preprint (which was not certified by peer review) is the author/funder, who has granted bioRxiv a license to display the preprint in perpetuity. It is made available under aCC-BY 4.0 International license.

Supplementary figures

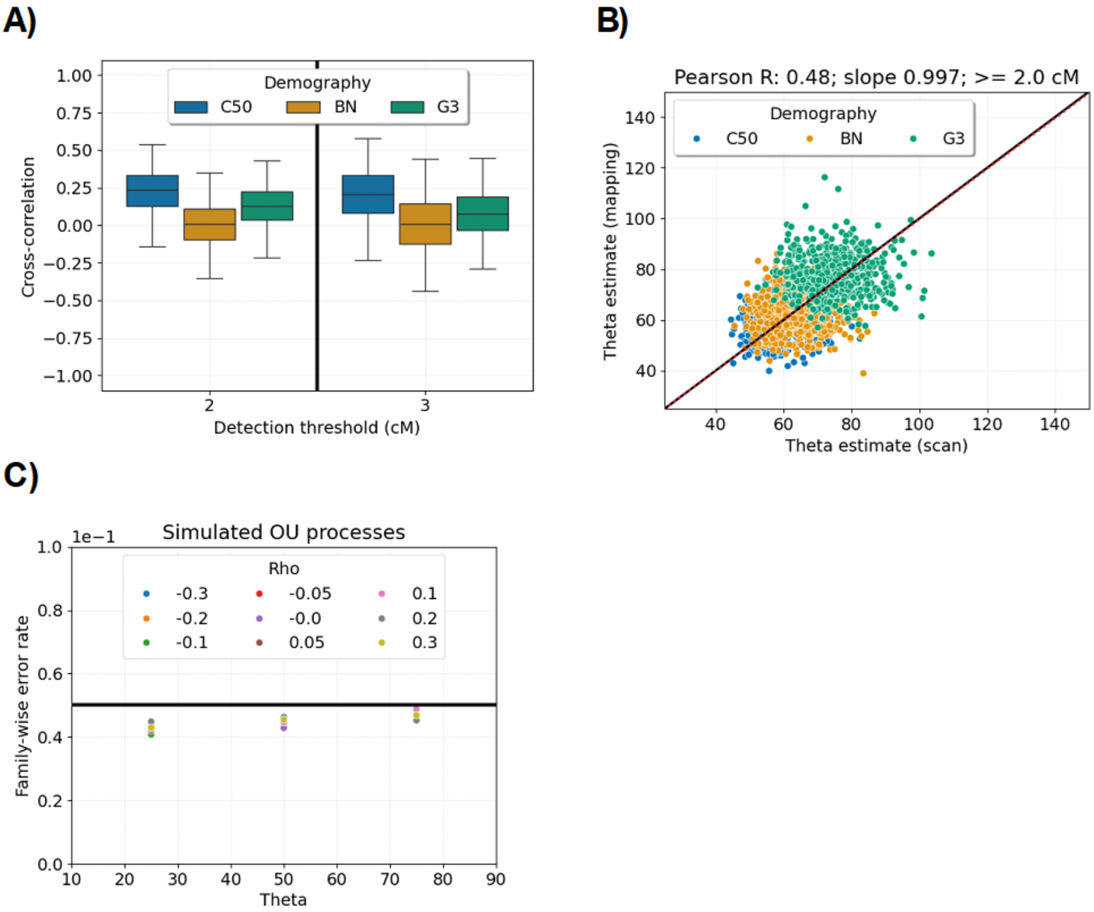

Figure S1: Robustness to nonzero cross-correlations. A) Boxplots show the 1st, 25th, 50th, 75th, and 99th percentiles of estimated correlations (y-axis) of case and control IBD rates with different IBD segment detection thresholds (x-axis). B) Estimates of the exponential decay parameter in the case-control scan (y-axis) are plotted versus the selection scan (x-axis). The simulated data come from a constant 50k (blue), population bottleneck (orange), and three phases of exponential growth (green) demographic scenarios. The case-control scan is performed by randomly assigning half of the 2500 samples to the case phenotype. C) The family-wise error rates (y-axis) of the standardized difference scan are plotted in terms of the exponential decay parameter θ (x-axis) and the cross-correlation ρ (legend). The desired family-wise error rate is 0.05 (indicated by the horizontal black line). The true Ornstein-Uhlenbeck process is simulated, and the true θ is used in calculating the discrete-spacing analytical threshold.

bioRxiv preprint doi: https://doi.org/10.1101/2025.07.03.663057; this version posted July 7, 2025. The copyright holder for this preprint (which was not certified by peer review) is the author/funder, who has granted bioRxiv a license to display the preprint in perpetuity. It is made available under aCC-BY 4.0 International license.

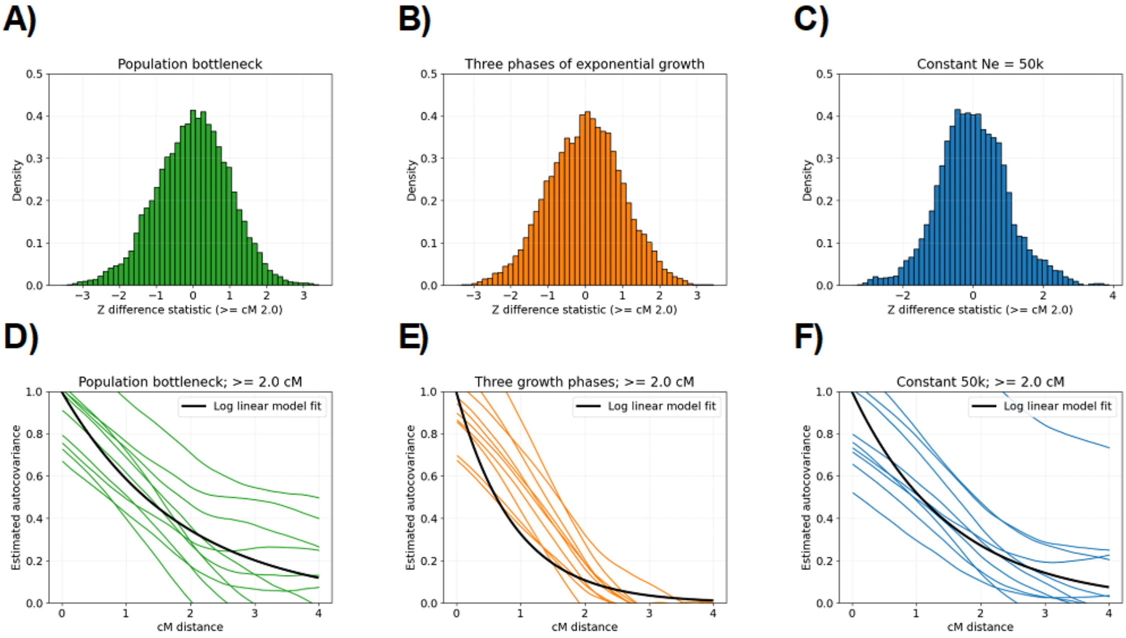

Figure S2: Distribution and autocovariance of standardized IBD rate differences in example data. A-C) The standardized IBD rate differences are plotted as histograms for population bottleneck (green), three phases of exponential growth (green), and constant 50k (blue) demographic simulations. Each histogram has 50 bins, and the x-axis ranges from -5 to 5. D-F) In estimating the exponential decay parameters for the IBD rate difference process, each colored line shows estimated autocovariances (y-axis) for different cM distances (x-axis) and a specific chromosome. The black lines represent the predicted autocovariances from the fitted Ornstein-Uhlenbeck processes using the estimated parameters $\hat{\theta}$. Each plot represents the results of one simulated example. The IBD segment detection threshold is ≥ 2.0 cM.

bioRxiv preprint doi: https://doi.org/10.1101/2025.07.03.663057; this version posted July 7, 2025. The copyright holder for this preprint (which was not certified by peer review) is the author/funder, who has granted bioRxiv a license to display the preprint in perpetuity. It is made available under aCC-BY 4.0 International license.

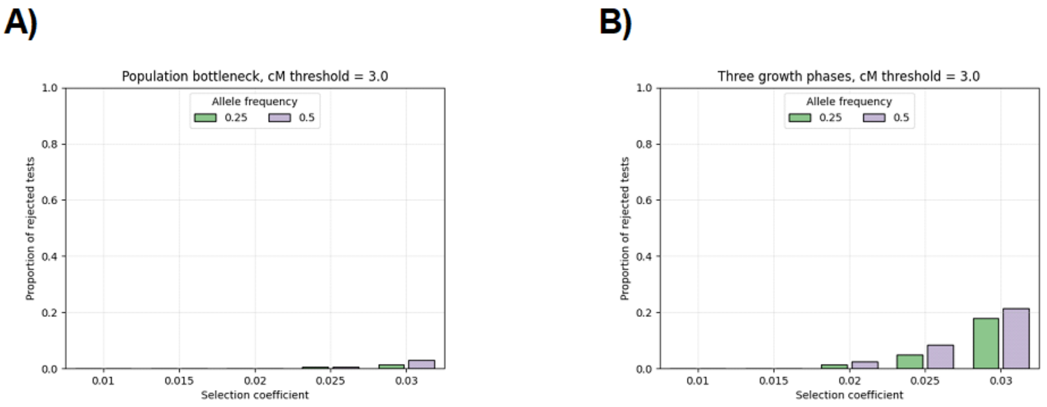

Figure S3: Proportion of false positives when strong positive selection is confounding and IBD segments are longer than 3.0 cM. Bar plots show the proportion of times that we reject the null hypothesis of the IBD rate difference scan in terms of the selection coefficient (x-axis) and the sweeping allele frequency (colors in legend). The demographic scenarios are A) population bottleneck and B) three phases of exponential growth. Each parameter combination is simulated 200 times. The significance threshold is based on the average threshold over all null simulations.

bioRxiv preprint doi: https://doi.org/10.1101/2025.07.03.663057; this version posted July 7, 2025. The copyright holder for this preprint (which was not certified by peer review) is the author/funder, who has granted bioRxiv a license to display the preprint in perpetuity. It is made available under aCC-BY 4.0 International license.

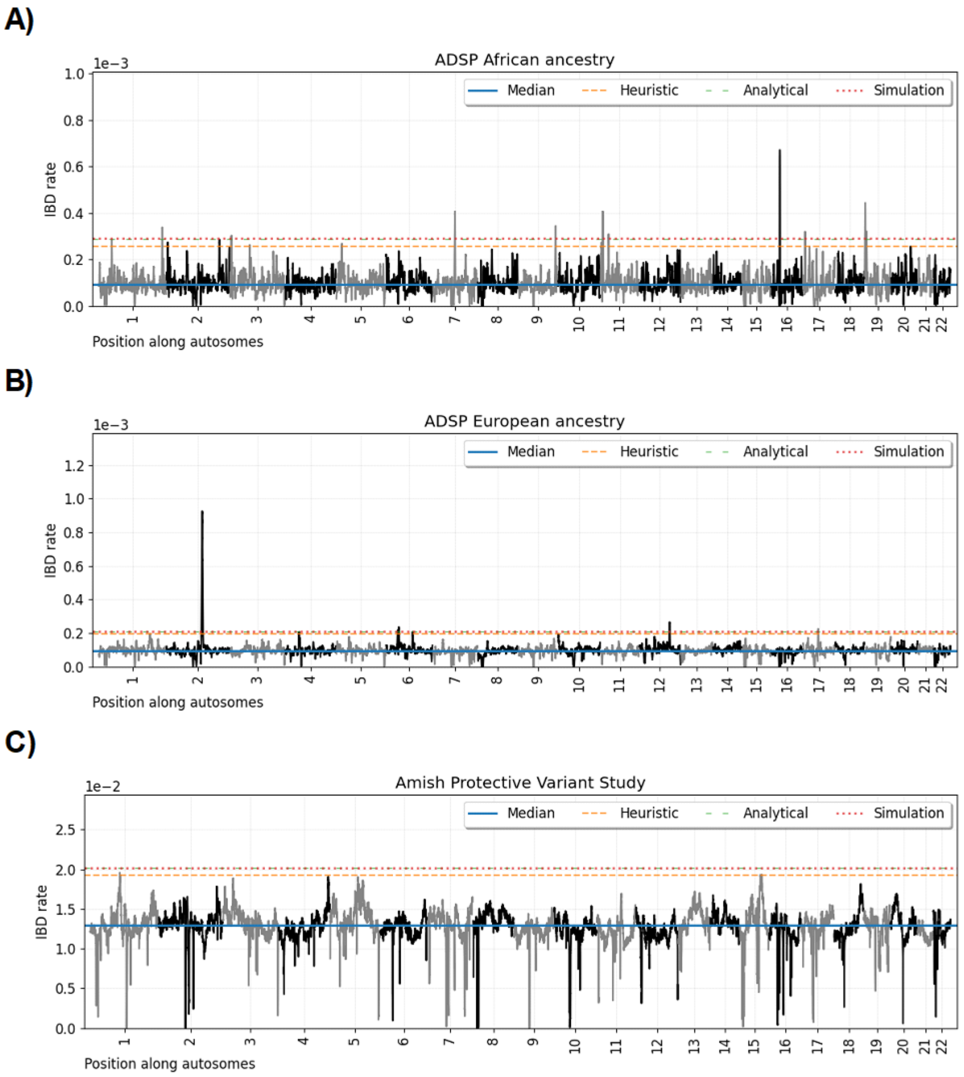

Figure S4: Genome-wide IBD rate scans for sample sets in the Alzheimer's Disease Sequencing Project. Line plots show IBD rates every 0.05 cM (y-axis) for base pair positions along twenty-two human autosomes. The data for each subplot is based on A) AFR ancestry, B) EUR ancestry, and C) Amish sample sets. Horizontal dashed lines show (blue) the autosome-wide median IBD rate, (orange) the heuristic threshold of four standard deviations above the median, (green) the discrete-spacing analytical threshold, and (red) the simulated-based threshold. The analytical and simulation-based thresholds differ by less than 5e-6. The IBD segment detection threshold is ≥ 2.0 cM.

bioRxiv preprint doi: https://doi.org/10.1101/2025.07.03.663057; this version posted July 7, 2025. The copyright holder for this preprint (which was not certified by peer review) is the author/funder, who has granted bioRxiv a license to display the preprint in perpetuity. It is made available under aCC-BY 4.0 International license.

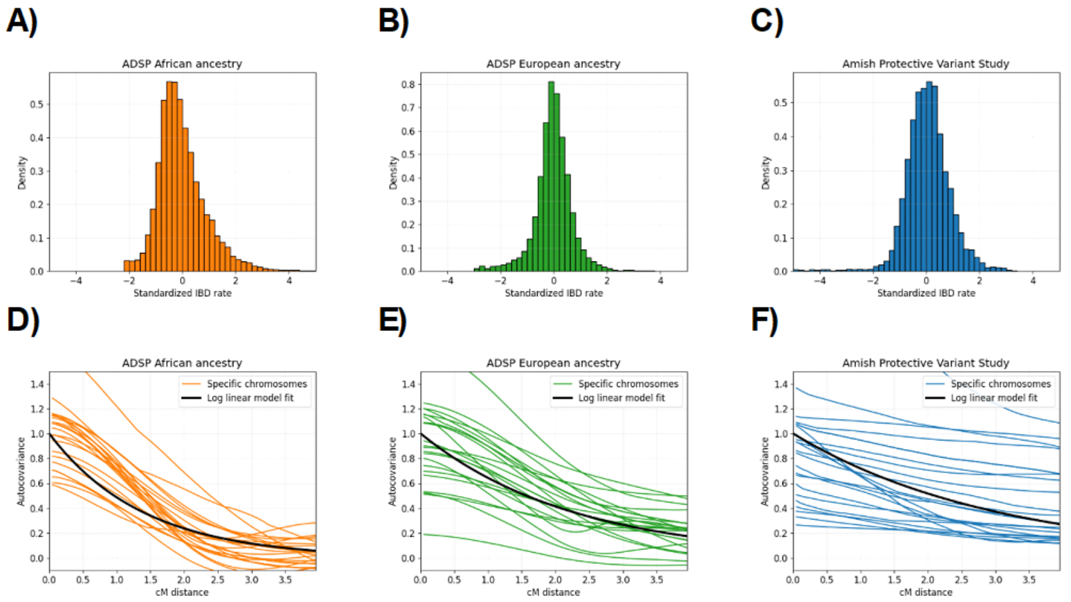

Figure S5: Distribution and autocovariance of IBD rates in selection scans. A-C) The standardized IBD rates are plotted as histograms for AFR ancestry (orange), EUR ancestry (green), and Amish (blue) sample sets. Each histogram has 50 bins, and the x-axis ranges from -5 to 5. D-F) In estimating the exponential decay parameters for the IBD rate process, each colored line shows estimated autocovariances (y-axis) for different cM distances (x-axis) and a specific chromosome. The black lines represent the predicted autocovariances from the fitted Ornstein-Uhlenbeck processes using the estimated parameters $\hat{\theta}$. Exponential decay estimates of the AFR, EUR, and Amish ancestry samples are 72, 44, and 33.

bioRxiv preprint doi: https://doi.org/10.1101/2025.07.03.663057; this version posted July 7, 2025. The copyright holder for this preprint (which was not certified by peer review) is the author/funder, who has granted bioRxiv a license to display the preprint in perpetuity. It is made available under aCC-BY 4.0 International license.

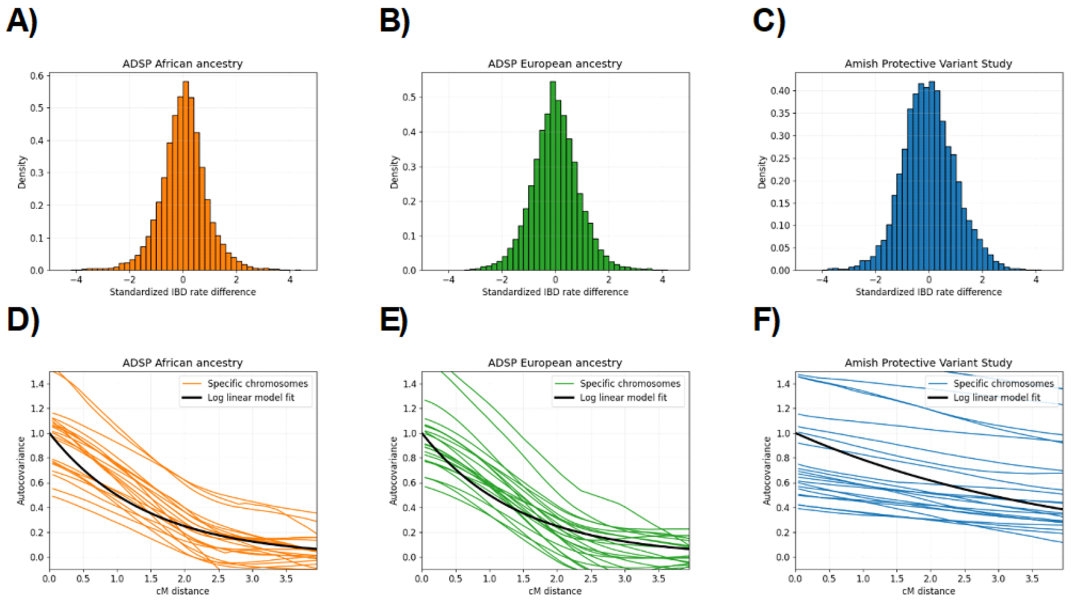

Figure S6: Distribution and autocovariance of standardized IBD rate differences in case-control scans. A-C) The standardized IBD rate differences are plotted as histograms for AFR ancestry (orange), EUR ancestry (green), and Amish (blue) sample sets. Each histogram has 50 bins, and the x-axis ranges from -5 to 5. D-F) In estimating the exponential decay parameters for the IBD rate difference process, each colored line shows estimated autocovariances (y-axis) for different cM distances (x-axis) and a specific chromosome. The black lines represent the predicted autocovariances from the fitted Ornstein-Uhlenbeck processes using the estimated parameters $\hat{\theta}$. Exponential decay estimates of the AFR, EUR, and Amish ancestry control samples are 70, 70, and 24.

bioRxiv preprint doi: https://doi.org/10.1101/2025.07.03.663057; this version posted July 7, 2025. The copyright holder for this preprint (which was not certified by peer review) is the author/funder, who has granted bioRxiv a license to display the preprint in perpetuity. It is made available under aCC-BY 4.0 International license.

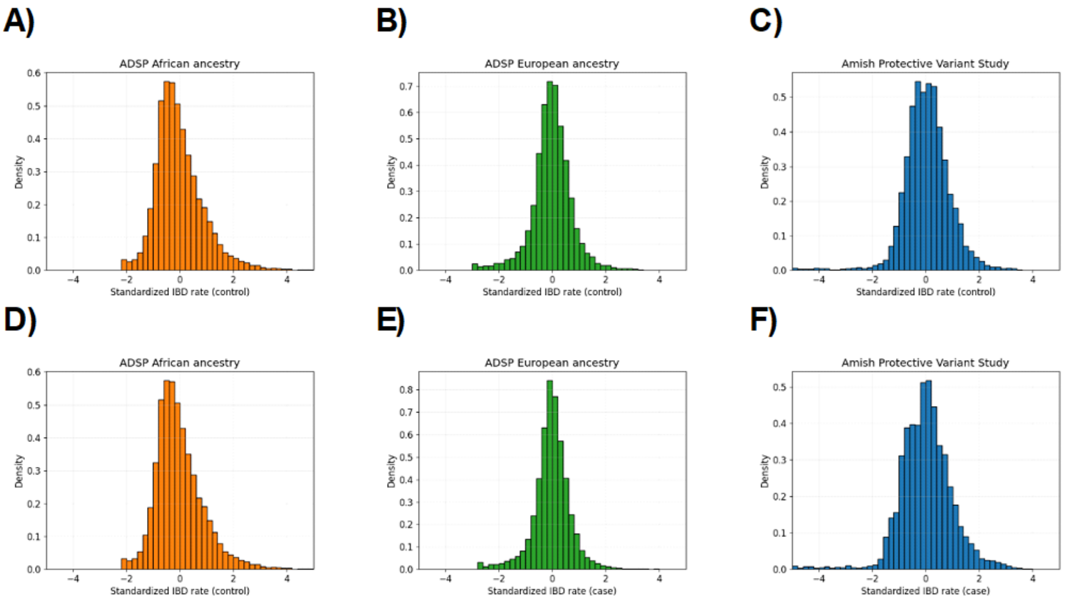

Figure S7: Histograms of standardized IBD rates in cases and controls. The standardized IBD rates $\geq$ 2.0 cM (x-axis) of controls A-C) and cases D-F) are shown for AFR (orange), EUR (green), and Amish (blue) sample sets. Each histogram has fifty bins, and the x-axes range from -5 to 5.

bioRxiv preprint doi: https://doi.org/10.1101/2025.07.03.663057; this version posted July 7, 2025. The copyright holder for this preprint (which was not certified by peer review) is the author/funder, who has granted bioRxiv a license to display the preprint in perpetuity. It is made available under aCC-BY 4.0 International license.

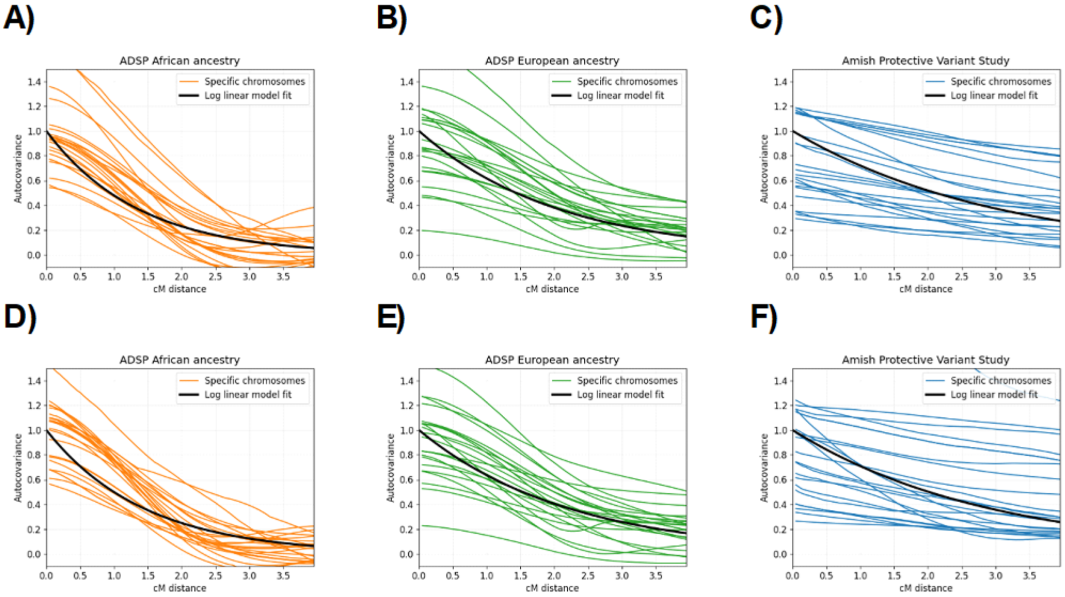

Figure S8: Estimating exponential decay parameter θ in the cases and controls separately. Each colored line shows estimated autocovariances (y-axis) for different cM distances (x-axis) and a specific chromosome. The black lines represent the predicted autocovariances from the fitted Ornstein-Uhlenbeck processes using the estimated parameters $\hat{\theta}$. The subplots A-C) and D-F) show results for case and control sample sets, respectively. The data for each subplot is based on AFR ancestry (orange), EUR ancestry (green), and C) Amish (blue) sample sets. Exponential decay estimates for the AFR, EUR, and Amish ancestry control samples are 73, 48, and 33, respectively. Exponential decay estimates of the AFR, EUR, and Amish ancestry control samples are 69, 45, and 34.

bioRxiv preprint doi: https://doi.org/10.1101/2025.07.03.663057; this version posted July 7, 2025. The copyright holder for this preprint (which was not certified by peer review) is the author/funder, who has granted bioRxiv a license to display the preprint in perpetuity. It is made available under aCC-BY 4.0 International license.

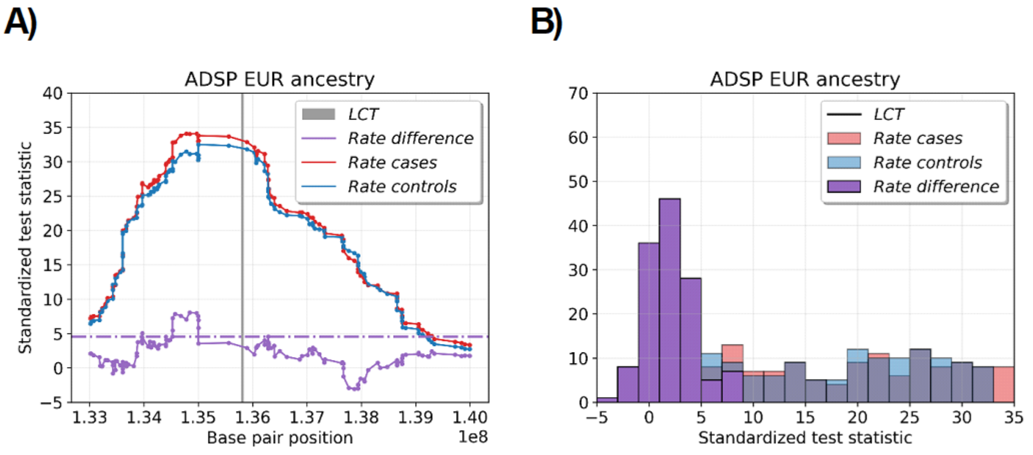

Figure S9: Randomized analysis of *LCT* gene. A) The line plot and B) the histogram show standardized test statistics every 0.05 cM for base pair positions around the *LCT* gene. The data is based on ≥ 2.0 cM IBD segments in all (purple), case (red), and control (blue) samples from the European ancestry cohort. We randomly assign half of the samples to be cases and the other half to be controls. A) The horizontal purple line shows analytical significance thresholds, and the gray span covers the *LCT* gene.

bioRxiv preprint doi: https://doi.org/10.1101/2025.07.03.663057; this version posted July 7, 2025. The copyright holder for this preprint (which was not certified by peer review) is the author/funder, who has granted bioRxiv a license to display the preprint in perpetuity. It is made available under aCC-BY 4.0 International license.

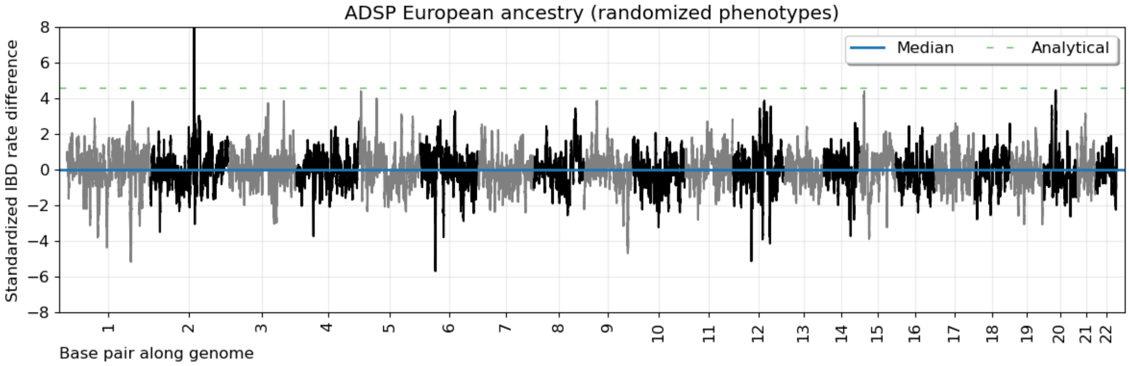

Figure S10: IBD rate difference scan with randomized binary phenotypes. Line plots show standardized IBD rate differences every 0.05 cM (y-axis) for base pair positions along twenty-two human autosomes. The data is based on $\geq$ 2.0 cM IBD segments in the EUR ancestry samples. We randomly assign half of the samples to be cases and the other half to be controls. Horizontal dashed lines show (blue) the autosome-wide median IBD rate, (orange) the heuristic threshold of four standard deviations above the median, (green) the discrete-spacing analytical threshold, and (red) the simulated-based threshold.

bioRxiv preprint doi: https://doi.org/10.1101/2025.07.03.663057; this version posted July 7, 2025. The copyright holder for this preprint (which was not certified by peer review) is the author/funder, who has granted bioRxiv a license to display the preprint in perpetuity. It is made available under aCC-BY 4.0 International license.

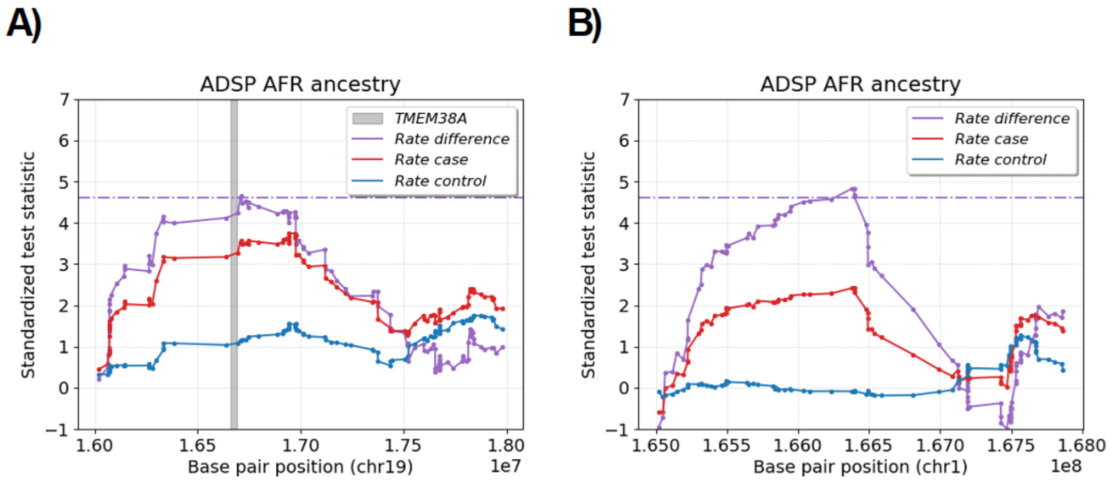

Figure S11: Two African ancestry-specific risk loci that are genome-wide significant in Alzheimer's disease case-control scan. The scatter plot shows the standardized test statistics (y-axis) by autosomal base pair position (x-axis) for two loci that are genome-wide significant. The test statistics are the IBD rate difference (purple), the IBD rate in cases (red), and the IBD rate in controls (blue). The horizontal purple lines are the genome-wide significance threshold in the case-control scan. A) The *TMEM38A* gene is shown in a gray shade.

bioRxiv preprint doi: https://doi.org/10.1101/2025.07.03.663057; this version posted July 7, 2025. The copyright holder for this preprint (which was not certified by peer review) is the author/funder, who has granted bioRxiv a license to display the preprint in perpetuity. It is made available under aCC-BY 4.0 International license.

Supplementary tables

Chr.	Final/Shared	Local ancestry	ADSP	1000 Genomes
1	1,539,963	1,029,049	25,458,292	5,759,060
2	1,651,632	1,094,233	27,774,708	6,088,599
3	1,390,811	931,292	22,996,880	4,983,186
4	1,402,424	942,234	22,204,088	4,875,466
5	1,279,500	848,993	20,725,489	4,536,820
6	1,248,708	849,142	19,470,667	4,315,218
7	1,141,316	766,489	18,281,152	4,137,255
8	1,074,540	713,733	17,555,631	3,886,223
9	856,040	572,599	13,946,989	3,165,514
10	978,395	656,688	15,340,230	3,495,474
11	960,064	643,210	15,560,762	3,423,342
12	929,495	627,283	15,030,264	3,332,789
13	701,957	472,702	11,145,065	2,509,180
14	634,641	424,983	10,201,558	2,290,401
15	573,934	385,469	9,290,105	2,109,286
16	597,814	394,846	10,119,456	2,362,362
17	535,494	354,442	8,960,527	2,073,625
18	550,839	371,816	8,739,032	1,963,846
19	425,089	285,259	6,691,868	1,670,693
20	444,471	296,539	7,136,626	1,644,385
21	262,067	177,931	4,003,489	1,002,753
22	267,777	181,668	4,180,531	1,066,557
Total	19,446,971	13,020,600	314,809,406	70,692,035

Table S1: Number of phased and ancestry-inferred variants. The variant counts are given for the shared and phased variants, the variants with local ancestry dosages, the initial unphased ADSP data, and the initial phased 1000 Genomes and HGDP data.

bioRxiv preprint doi: https://doi.org/10.1101/2025.07.03.663057; this version posted July 7, 2025. The copyright holder for this preprint (which was not certified by peer review) is the author/funder, who has granted bioRxiv a license to display the preprint in perpetuity. It is made available under aCC-BY 4.0 International license.

**Software**	**Parameter**	**Value**
`Beagle 5.4`	impute	false
	window	5.0
`flare`	min-maf	0.005
	min-mac	10
	gen	10
	probs	false
`hap-ibd`	min-seed	1.0
	min-extend	0.2
	min-output	2.0
	min-mac	10
	max-gap	1000
	min-markers	100

Table S2: Parameter settings for phasing, local ancestry inference, and relatedness inference.

bioRxiv preprint doi: https://doi.org/10.1101/2025.07.03.663057; this version posted July 7, 2025. The copyright holder for this preprint (which was not certified by peer review) is the author/funder, who has granted bioRxiv a license to display the preprint in perpetuity. It is made available under aCC-BY 4.0 International license.

**Superpopulation**	**Subpopulation**	**Sample size**
African (AFR)	GWD	176
	LWK	97
	YRI	174
	Total	447
East Asian (EAS)	CHB	103
	CHS	163
	JPT	102
	Total	368
European (EUR)	CEU	174
	GBR	90
	TSI	103
	Total	367
South Asian (SAS)	BEB	131
American (AMR)	MXL	14
	PEL	88
	Total	102

Table S3: Sample sizes for reference panels. The abbreviations are Gambian in Western Division, The Gambia (GWD), Luhya in Webuye, Kenya (LWK), Yoruba in Ibadan, Nigeria, Han Chinese in Beijing, China (CHB), Southern Han Chinese (CHS), Japanese in Tokyo, Japan (JPT), Utah residents with Western and Northern European ancestry (CEU), British in England and Scotland (GBR), Toscani in Italy (TSI), Bengali in Bangladesh (BEB), Mexican Ancestry in Los Angeles, California (MXL), and Peruvian in Lima, Peru (PEL).

bioRxiv preprint doi: https://doi.org/10.1101/2025.07.03.663057; this version posted July 7, 2025. The copyright holder for this preprint (which was not certified by peer review) is the author/funder, who has granted bioRxiv a license to display the preprint in perpetuity. It is made available under aCC-BY 4.0 International license.

Software	Parameter	Value
`hap-ibd` (EUR & AFR)	min-seed	1.0
	min-extend	0.2
	min-output	2.0
	min-maf	0.40
`hap-ibd` (Amish)	min-seed	0.5
	min-extend	0.2
	min-output	1.0
	min-maf	0.01
`ibd-ends`	err	1.0e-4
	quantiles	0.5
	min-maf	0.001
Selection and case-control scans	step_size_cm	0.05
	scan_cutoff	2.0
	confidence_level	0.05
	num_sims	500
	cm_gap	0.5
	outlier_cutoff	4.0
	auto_covariance_length	4.0
Selection scan	covered_cm_region	0.50

Table S4: Parameter settings for selection and case-control scans. If not specified, default settings for sequence data are otherwise used in `hap-ibd` and `ibd-ends`. For the African and European ancestry analyses, we use `hap-ibd` for candidate segments and `ibd-ends` for refining the segment endpoints. For the Amish analysis, we only use `hap-ibd` with the sequence data settings. The `ibd-ends` error rate is set to 1e-4 based on initial estimates from preliminary analyses of chromosomes 20 to 22.

bioRxiv preprint doi: https://doi.org/10.1101/2025.07.03.663057; this version posted July 7, 2025. The copyright holder for this preprint (which was not certified by peer review) is the author/funder, who has granted bioRxiv a license to display the preprint in perpetuity. It is made available under aCC-BY 4.0 International license.

Study	Chr	Max rate (1e-4)	Size (cM)	Position (Mb)	Genes
EUR	2	9.25	6.55	135.00 (132.88-139.25)	*LCT*
	12	2.64	2.15	113.09 (111.19-113.55)	*OAS1-2-3*
	6	2.36	1.45	33.99 (33.68-34.44)	*MHC*
	17	2.25	1.40	37.08 (36.93-37.76)	*HNF1B*
	6	2.17	0.55	25.00 (24.95-25.33)	*MHC*
AFR	16	6.71	2.65	17.24 (16.75-18.01)	*XYLT1*
	19	4.44	2.45	17.25 (15.51-20.98)	.
	7	4.07	2.00	80.70 (80.13-81.34)	*SEMA3C*
	11	4.06	2.05	5.75 (5.27-6.32)	*HBB*
	9	3.45	1.25	134.60 (134.55-134.66)	.
	1	3.38	1.45	240.54 (240.45-240.64)	.
	19	3.21	1.20	3.09 (3.02-3.14)	.
	17	3.20	1.10	3.87 (3.80-3.93)	.
	11	3.09	0.90	19.94 (19.89-20.00)	.
	3	3.03	1.10	3.09 (3.04-3.09)	.

Table S5: Loci detected in selection scans of the Alzheimer's Disease Sequencing Project data. We report loci where identity-by-descent (IBD) rates exceed the discrete-spacing analytical thresholds for the AFR ancestry and EUR ancestry samples. The maximum IBD rate is given for each locus. Physical positions for the location of the maximum IBD rate in loci where excess IBD rates span more than 0.5 cM are shown in megabases (Mb). The sizes of the excess IBD regions are shown in centiMorgans (cM). Annotated genes or gene complexes are discussed in the main text. The IBD segment detection threshold is 2.0 cM.

bioRxiv preprint doi: https://doi.org/10.1101/2025.07.03.663057; this version posted July 7, 2025. The copyright holder for this preprint (which was not certified by peer review) is the author/funder, who has granted bioRxiv a license to display the preprint in perpetuity. It is made available under aCC-BY 4.0 International license.

Ancestry	Gene	Target	High Risk	Protein DE	RNA DE
AFR	*DCDC2C*	No	—	—	—
	COLEC11	No	No	—	TCX
	ICE1	No	No	—	CBE
	ADAMTS6	No	No	—	PCC
	TMEM38A	No	Yes	Yes	FP,IFG,PCC,PHG,STG,TCX
EUR	*NBAS*	Yes	Yes	No	ACC,DLPFC,PHG,TCX
Amish	*ASTN1*	Yes	Yes	Yes	PHG,TCX
	BRINP2	Yes	Yes	No	CBE,DLPFC,IFG,PCC,PHG,STG,TCX

Table S6: Annotations from Agora web resource of genes in risk loci. Each gene is located within or less than a few hundred kilobases of genome-wide significant loci in the case-control scans of three ancestry cohorts. Columns concern if the gene is a nominated therapeutic target (Target), has a multi-omic risk score greater than 3.80 (High Risk), has evidence of differential protein expression in post-mortem AD individuals, or has RNA differential expression (DE) in a brain region. The brain regions are the anterior cingulate cortex (ACC), cerebellum (CBE), dorsolateral prefrontal cortex (DLPFC), frontal pole (FP), inferior frontal gyrus (IFG), posterior cingulate cortex (PCC), parahippocampal gyrus (PHG), superior temporal gyrus (STG), and temporal cortex (TCX). Horizontal lines indicate that there is no data available for that measurement.
